# Supplementary material for: Promoting smoking cessation and preventing relapse to tobacco use following a smoke-free mental health inpatient stay (SCEPTRE feasibility study): a multicentre randomised controlled feasibility study protocol
Source: BMJ Open. 2025 Jun 19;15(6):e094441. doi: 10.1136/bmjopen-2024-094441 (PMC12182159; doi:10.1136/bmjopen-2024-094441)
Supplement: Supplementary file 3 [file bmjopen-2024-094441.reviewer_comments.pdf]

PEER REVIEW HISTORY

BMJ Open publishes all reviews undertaken for accepted manuscripts. Reviewers are asked to complete a checklist review form and are provided with free text boxes to elaborate on their assessment. These free text comments are reproduced below.

ARTICLE DETAILS

Title (Provisional)

Promoting Smoking Cessation and Preventing Relapse to Tobacco Use following a smokefree mental health in-patient stay (SCEPTRE feasibility study): a multi-centre randomised controlled feasibility study protocol.

Authors

Petersen Williams, Petal; Huddlestone, Lisa; Shoesmith, Emily; Brady, Samantha; Mitchell, Alex; Exley, Victoria; Wiggins, Fraser; Sinclair, Lesley; Pervin, Jodi; Horspool, Michelle; Leahy, Moira; Paul, Claire; Colley, Lesley; Shahab, Lion; Watson, Judith; Hewitt, Catherine; Hough, Simon; Britton, John; Coleman, Tim; Gilbody, Simon; Parrott, Steve; Galdas, Paul; Russell, Gregor; Coventry, Peter A; Ratschen, Elena

---

VERSION 1 - REVIEW

**Reviewer**	**1**
**Name**	**OConnell, Nicola.**
**Affiliation**	**Health Service Executive**
**Date**	**30-Oct-2024**
**COI**	**None**

This is a well written and interesting protocol in an under-researched area. I have minor comments in the attachment. My main concern is the broad nature of the inclusion criteria, there is no plan to select patients based on assessment of severity and the intervention itself appears to be aimed at both maintaining quitting as well as reducing cigarette smoking. On the latter, it feels odd that an intervention, can do both well. The issues around contamination of the control group and the possibility of a future cluster randomised design are well made, but I would suggest randomsiation at a Trust level, rather than a ward level to account for Trust smokefree policies.

Line 157: Are PPI members paid for their involvement?

Line 175: Change 'mental health' to upper case 'Mental Health Trusts'

Line 174: The paper doesn't give a sense of the kind of inpatient settings where recruitment will take place e.g. how many different settings will be included in each of the 8 Trusts? Will there be even

distribution of inpatient settings across the Trusts to avoid over-representation of some e.g. in very poor or wealthy catchment areas. Will specialist inpatient settings be included e.g. a mother and baby inpatient unit?

Line 174: How were the 8 Trusts chosen for recruitment and why?

Line 176: The paper says “Trusts must be committed to, and working towards full implementation of smokefree policy”, does that mean that some of the 8 included Trusts don’t have a full smokefree policy? If so, the patients won’t have be exposed to a period of enforced abstinence via the hospital’s policy.

Line 179: The protocol states that patients who are both smokers and non-smokers at discharge will be included in the study. Does this not have implications for the kind of intervention that is/can be delivered – one that has to either support participants to maintain their quit behaviour or encourage participants to give up and/or reduce smoking? How can an intervention do all of these things at the same time and are their implications for measurement of the behavioural outcome?

Line 179: Will patients with co-morbid drug and alcohol addictions be included? They may have had previous exposure to quit attempts through recovery services that could complicate their inclusion

Line 198: It’s not clear who the ‘Trust-based researchers’ are. Are they employees of the Trusts, or are they employed by the University of York but physically based in the Trusts? How many researchers will be employed/based in each Trust? This will be useful info

Line 212: Spell out ‘PIS’

Line 230: Having a second follow-up period that is variable will affect the feasibility results, as it will be easier to follow up participants at 4 months than 6 months. Why not choose a 4-month follow-up point to avoid the variability?

Line 227: From reading the study, the intervention seems predicated on the fact participants will receive it outside the hospital setting, after discharge. What about hospitalised patients who are recruited to the study and randomised, but their inpatient stay turns out to be longer than three months and 4-6 months? How will their data be treated in the analysis? Will they just not receive the intervention and their data not analysed – how will this affect the sample size and recruitment goal?

Line 262: ‘Trust’ written with uppercase and without in cases. Use uppercase throughout.

Line 271: Explain ‘YTU’ acronym here

Line 275: No justification is given as to why the trial statistician won’t be blinded.

Line 390: The protocol doesn’t mention whether attempts will be made to record the reasons participants drop out of the trial.

Line 400: Consider using the PRECIS-2 tool to assess how pragmatic/explanatory the trial is – useful information if considering a later definitive trial

Line 412: If clusters are considered for a definitive trial, are Trusts not a better unit of randomisation to account for Trust-wide smoking policies? Can the authors explain why they would use wards and not Trusts in such a scenario?

Line 501: 15 days is a long time to report serious adverse events to the REC and sponsor. It is also inadvisable to wait until the next PSC and DMEC before reporting any such events as they may

decide the effect of the trial is so serious that the trial needs to stop. This is unlikely, but it is important that any such issues are reviewed quickly in order for broader decisions around the trial's continuation to be made.

Line 516: Is there a concern that there aren't stricter inclusion criteria in this trial. There will be a wide variety of mental health conditions of patients, but there don't seem to be any inclusion criteria based on severity, aside from not recruiting from PICUs. Treatment engagement with mental health patients is difficult and there may be some bias introduced here whereby less severely ill patients will be recruited to the study reducing generalisability, but there may be higher dropout amongst the more severely ill patients. This may be somewhat accounted for by randomisation, but can the authors comment on having such a wide inclusion criteria regarding illness severity?

Line 517: Related to this is the issue regarding including both people who want to reduce their cigarette intake and people who want to maintain their quitting. These are qualitatively different outcomes and it is surprising that the intervention has been developed that can address both. Similarly to the point above, can the authors comment on why two behavioural change outcomes are included, and why no smoking frequency criteria is included, as it would be easier for a person to quit if smoking one or two cigarettes a day compared to a smoker of 20 cigarettes a day. The heterogeneity of the sample due to these wide inclusion criteria is somewhat concerning.

**Reviewer**	**2**
**Name**	**Saxon, Andrew J.**
**Affiliation**	**University of Washington**
**Date**	**22-Feb-2025**
**COI**	**None**

This manuscript describes the protocol for what is essentially a randomized, pilot feasibility trial on a crucially important topic, an intervention to help patients who smoke tobacco and are admitted to a smokefree psychiatric inpatient unit and must stop tobacco use during the inpatient stay to extend tobacco abstinence into the post-discharge phase.

For the most part the protocol seems well thought out and well designed and clearly conveyed in the manuscript. The plan to do this feasibility trial to inform a future definitive trial deserves admiration because it will undoubtedly lead to a more highly informed and successfully performed ultimate protocol.

However, the manuscript does have a serious flaw in that the discussion section is quite inadequate. Although the design decisions made seem reasonable on their face, several decisions could have been made differently, and an expanded discussion section could address the pros and cons of various design aspects and why the specific methods were chosen.

Some obvious examples include the following (though this list may not be exhaustive):

1. While the trial is a randomized test of the intervention, there are apparently no plans to compare outcomes directly between the experimental and control conditions. It is understood that the sample size of the pilot is unlikely to have sufficient power to show statistical separation between the conditions. Many pilot trials do at least calculate effect sizes that can help support the power analysis for the larger, definitive trial. Why is that maneuver not part of the plan for this protocol?

2. It is not clear why nicotine replacement therapy or electronic cigarettes are the only pharmacological options, especially since varenicline is now seen as somewhat superior in efficacy to nicotine replacement therapy. On this topic electronic cigarettes apparently are more controversial among U.S. tobacco treatment experts than they are in the U.K. even though they look good in randomized trials as a cessation pharmacotherapy and probably pose less health risks than combustible tobacco. Nevertheless, they probably have more health risks than nicotine replacement so the decision to use them should be justified. Also, more detail about the nicotine replacement would be helpful. Will the protocol allow combination of a long-acting form of replacement (i.e. transdermal nicotine) with a short-acting form (e.g. gum or lozenge)?

3. Many patients admitted to inpatient psychiatric units suffer from psychosis and/or mania, yet the protocol has no instruments to assess such symptomatology. Why not? Such syndromes could be affected by tobacco cessation treatments.

4. Many trials that study a behavioral intervention use actual recordings of treatment sessions to assess fidelity. This protocol relies mainly on self-report by the clinicians delivering the intervention for fidelity assessments. What prompted the decision not to record the sessions? Additionally, in regards to line 332, What would “random fidelity checks” consist of?

5. Since breath carbon monoxide is the primary outcome measure and since new technologies have the ability to do remote carbon monoxide testing and since most of the interventions will be delivered remotely anyway, why was remote breath carbon monoxide testing not considered? It would likely reduce the amount of missing data in regard to the primary outcome. On this topic the manuscript alludes to the fact that the optimal breath carbon monoxide level to determine abstinence may be uncertain and that the chosen threshold of 10 parts per million may be too high and might need to be lowered. What, then, led to the decision to choose this somewhat high threshold?

An additional important point is that person centered, non-stigmatizing language should be used throughout the protocol. “Patients who smoke tobacco” rather than “smokers” is more appropriate. The terms “smoker” or “smokers” should be completely eliminated from the manuscript.

Finally, the acronyms “PIS” and “APEASE” require definitions.

VERSION 1 - AUTHOR RESPONSE

**Reviewer 1: Dr. Nicola. OConnell, Health Service Executive**	
**Comments**	**Response**
My main concern is the broad nature of the inclusion criteria, there is no plan to select patients based on assessment of severity and the intervention itself appears to be aimed at both maintaining quitting as well as reducing cigarette smoking. On the latter, it feels odd that an intervention, can do both well.	We appreciate the reviewer's concern regarding the broad inclusion criteria. We would like to point out that the primary goal of the intervention is to support ALL patients who are admitted as smokers to a unit to prevent relapse to pre-admission smoking behaviours after discharge. The rationale is laid out in detail in the background section. Our approach to inclusion is intentional and aligns with real-world clinical tobacco dependence treatment practice, where individuals with varying levels of tobacco dependence and motivation to quit are supported within mental health services. Given the high smoking prevalence among individuals with mental health conditions, we aimed for an inclusive approach to reflect the diverse patient population in acute inpatient settings. Many patients fluctuate in their readiness to quit, and a strict severity of dependence-based selection would have limited recruitment and generalisability and not been useful in terms of existing evidence. While the intervention supports both smoking reduction and quitting, these are not mutually exclusive aims. The intervention is designed to be flexible, supporting both smoking reduction and cessation, as reduction has been shown to be frequently a stepping stone towards quitting. The tobacco-related inequalities in this population are so vast that focusing on harm reduction represents a worthwhile and important goal, and our intervention was designed to be flexibly applicable to all individuals who express an interest in addressing their smoking, be it by quitting or reducing, post discharge.<br>Our process evaluation will explore how participants engage with the intervention based on their smoking status and motivation, informing future refinements.
The issues around contamination of the control group and the possibility of a future cluster randomised design are well made, but I would suggest randomisation at a Trust	Thank you for the helpful suggestion. It is important to know that the way in which smokefree policies are implemented in UK mental health Trusts tends to vary considerably by ward and location (e.g.,

level, rather than a ward level to account for Trust smokefree policies.	some wards based in rural settings; others in high rise urban hospitals), and randomisation by Trust would have had even greater implications on the required sample size than randomisation by ward. This feasibility study is being conducted to assess, amongst other aspects, whether the randomisation approach is suitable (see above).
Line 157: Are PPI members paid for their involvement?	PPI members do get paid for their involvement and it's £25 per hour - either via bank transfer or vouchers (depending on their preference). This is in line with NIHR/UoY policy. We have indicated that they are paid in the manuscript.
Line 175: Change 'mental health' to upper case 'Mental Health Trusts'	This has been corrected.
Line 174: The paper doesn't give a sense of the kind of inpatient settings where recruitment will take place e.g. how many different settings will be included in each of the 8 Trusts? Will there be even distribution of inpatient settings across the Trusts to avoid over-representation of some e.g. in very poor or wealthy catchment areas. Will specialist inpatient settings be included e.g. a mother and baby inpatient unit?	Recruitment will take place in 'acute adult mental health inpatient wards' across up to eight NHS Trusts. This does by definition not include specialist inpatient settings, such as mother and baby units, as our focus is on adult mental health services. Trusts selection is based on expressions of interest rather than a formal even distribution of inpatient settings. We have clarified this in the text. However, we aimed for diversity in participating sites to ensure a mix of different geographic and socioeconomic contexts. The feasibility study will allow us to assess any potential imbalances and consider adjustments for a future definitive trial.
Line 174: How were the 8 Trusts chosen for recruitment and why?	Please see comment above.
Line 176: The paper says "Trusts must be committed to, and working towards full implementation of smokefree policy", does that mean that some of the 8 included Trusts don't have a full smokefree policy? If so, the patients won't have be exposed to a period of enforced abstinence via the hospital's policy.	All participating Trusts are required to have a smokefree policy in place, in line with national guidance. The wording in the manuscript reflects that while all Trusts are implementing smokefree policies, there may be variations in the degree of enforcement and support available across sites. Policy adherence can differ depending on operational and cultural factors within each Trust. However, all patients recruited will have been admitted to wards where a smokefree policy is in effect, ensuring they have experienced a period of enforced abstinence during their stay. We will document and report any variations in policy implementation as part of the study.

Line 179: The protocol states that patients who are both smokers and non-smokers at discharge will be included in the study. Does this not have implications for the kind of intervention that is/can be delivered – one that has to either support participants to maintain their quit behaviour or encourage participants to give up and/or reduce smoking? How can an intervention do all of these things at the same time and are their implications for measurement of the behavioural outcome?	The intervention is designed to help people prevent return to pre-admission smoking behaviours. They may have quit while on the ward or managed to cut down considerably while on the ward. The intervention promotes smoking cessation while acknowledging that for many people, cutting down is a first step towards quitting. This is a low threshold, service-orientated intervention designed to support as many potentially susceptible smokers with mental illness as possible, using a theory- and evidence-based, tailored approach. The intervention was designed to be flexible and patient-centred, recognising that individuals in mental health settings have diverse smoking histories, motivations, and quitting trajectories. Supporting both maintaining abstinence and reducing smoking is in line with behavioural change models, where reduction can serve as a pathway to cessation.<br>To ensure appropriate tailoring, the intervention includes personalised goal-setting sessions, where participants can set objectives based on their current smoking status and motivation. Those who have quit can receive relapse prevention support, while those still smoking can access guidance on reduction or cessation.<br>Regarding measurement, our primary feasibility outcomes focus on recruitment, retention, and data collection processes. Smoking-related outcomes, including abstinence, reduction, and relapse, will be carefully tracked to capture the full spectrum of smoking behaviour change. Our process evaluation will further explore how participants engage with the intervention based on their smoking status at discharge, informing refinements for a future full trial.
Line 179: Will patients with co-morbid drug and alcohol addictions be included? They may have had previous exposure to quit attempts through recovery services that could complicate their inclusion	We are testing the effectiveness of an approach aimed at supporting mental health inpatients to prevent return to pre-admission smoking behaviours (see SCEPTRE acronym/title, and background section). Patients' history and context are secondary to this primary goal. Patients with co-morbid drug and alcohol addictions will not be specifically excluded, and our aim is to reflect the diversity of smokers in mental

	health inpatient settings. While some may have had prior exposure to quit attempts through recovery services, this variability is expected. We will collect data on participants' smoking history, including any previous quit attempts, to account for these factors in our analysis.
Line 198: It's not clear who the 'Trust-based researchers' are. Are they employees of the Trusts, or are they employed by the University of York but physically based in the Trusts? How many researchers will be employed/based in each Trust? This will be useful info	The Trust-based researchers are employees of the participating NHS Trusts and are part of the Research and Development (R&D) teams within each Trust whose role is facilitating research activities, including identifying potential participants, supporting recruitment, and collecting data. The number of researchers involved will vary by Trust, depending on local capacity and resources, but each participating Trust will have designated R&D team members supporting the study. We have included these details.
Line 212: Spell out 'PIS'	This has been done.
Line 230: Having a second follow-up period that is variable will affect the feasibility results, as it will be easier to follow up participants at 4 months than 6 months. Why not choose a 4-month follow-up point to avoid the variability?	The variable follow-up period became necessary because we extended the recruitment period to improve participant enrolment while ensuring the study could conclude within the funded timeframe. This approach allowed us to maximise recruitment without extending the overall study duration. While we acknowledge that follow-up at 4 months may be easier than at 6 months, retaining flexibility in the second follow-up period enables us to collect as much feasibility data as possible across a wider timeframe. We will account for any differences in follow-up duration when interpreting feasibility outcomes.
Line 227: From reading the study, the intervention seems predicated on the fact participants will receive it outside the hospital setting, after discharge. What about hospitalised patients who are recruited to the study and randomised, but their inpatient stay turns out to be longer than three months and 4-6 months? How will their data be treated in the analysis? Will they just not receive the intervention and their data not analysed – how will this affect the sample size and recruitment goal?	It is correct that the intervention is designed to be delivered post-discharge. To ensure participants can engage with the intervention as intended, one of our inclusion criteria is that they must have a planned discharge within the Trust's catchment area at the time of recruitment. Patients who experience an unexpectedly prolonged inpatient stay would not be able to receive the intervention as intended and, therefore, would not be recruited into the study. While this may pose a recruitment challenge, the inclusion criterion helps ensure that the intervention is

	delivered as designed and that feasibility outcomes are assessed in the appropriate population. Again, the feasibility study will help us gauge whether this approach is viable.
Line 262: 'Trust' written with uppercase and without in cases. Use uppercase throughout.	This has been corrected throughout.
Line 271: Explain 'YTU' acronym here	This has been done.
Line 275: No justification is given as to why the trial statistician won't be blinded.	The trial statistician will not be blinded due to the nature of the feasibility study, which focuses on recruitment, retention, and data collection processes rather than formal hypothesis testing of intervention efficacy. Blinding would add complexity without significantly impacting the integrity of the feasibility outcomes. However, to minimise potential bias, the statistician will follow a pre-specified analysis plan. If a full trial is conducted, the trial statistician will be blinded as appropriate. This justification has been included in the text.
Line 390: The protocol doesn't mention whether attempts will be made to record the reasons participants drop out of the trial.	We will attempt to record the reasons for participant dropout wherever possible. If a participant withdraws, we will document any reasons they may provide, although we recognise that some participants may choose not to disclose this information. This will help us understand potential barriers to retention and inform the design of a future full trial. We have added this information to the text.
Line 400: Consider using the PRECIS-2 tool to assess how pragmatic/explanatory the trial is – useful information if considering a later definitive trial	Thank you for this helpful suggestion. The trial has now been conducted, and we are unable to include the tool at this late a stage of our work, but we will bear it in mind for future studies.
Line 412: If clusters are considered for a definitive trial, are Trusts not a better unit of randomisation to account for Trust-wide smoking policies? Can the authors explain why they would use wards and not Trusts in such a scenario?	This is a very good point. It is important to note that smokefree policy implementation practices tend to vary considerably between wards in each Trust. Clustering by Trust would have very substantial implications for the sample size calculation and probably render the conduct of a fully powered trial impossible in the UK.
Line 501: 15 days is a long time to report serious adverse events to the REC and sponsor. It is also inadvisable to wait until the next PSC and DMEC before reporting any	We understand the concern regarding the 15-day timeline for reporting serious adverse events. However, as per the Health Research Authority (HRA) guidance, 15 days is

such events as they may decide the effect of the trial is so serious that the trial needs to stop. This is unlikely, but it is important that any such issues are reviewed quickly in order for broader decisions around the trial's continuation to be made.	considered standard practice for non-clinical trials of investigational medicinal products (non-CTIMPs). https://www.hra.nhs.uk/approvals-amendments/managing-your-approval/safety-reporting/<br><br>Although the next PSC and DMEC meeting may not immediately address such events, there are established protocols for expedited reporting and review when the severity or potential risks of a SAE could affect the trial's continuation.
Line 516: Is there a concern that there aren't stricter inclusion criteria in this trial. There will be a wide variety of mental health conditions of patients, but there don't seem to be any inclusion criteria based on severity, aside from not recruiting from PICUs. Treatment engagement with mental health patients is difficult and there may be some bias introduced here whereby less severely ill patients will be recruited to the study reducing generalisability, but there may be higher dropout amongst the more severely ill patients. This may be somewhat accounted for by randomisation, but can the authors comment on having such a wide inclusion criteria regarding illness severity?	Our goal is to be as inclusive and real-life-orientated as possible. Our broad inclusion criteria are intentional, as they reflect the diverse population of smokers in acute mental health inpatient settings and align with real-world clinical practice. Restricting by illness severity could limit recruitment and reduce the applicability of findings to routine care. While we acknowledge that engagement in research can be more challenging for individuals with more severe illness, our approach allows us to assess the feasibility of recruiting and retaining a representative sample.<br>We have excluded patients in PICUs due to their high levels of acute distress and usually limited capacity to engage in research. Beyond this, all eligible patients who meet the inclusion criteria, including those with varying illness severity, will be approached. Randomisation should help balance differences in illness severity between groups, and we will collect baseline clinical and demographic data to explore potential differences in engagement and retention across participants. Understanding these factors will help refine recruitment strategies for a future full trial. This trial has been co-designed with a sizeable team of psychiatrists and other mental health clinicians. We have added additional information regarding this in the discussion.
Line 517: Related to this is the issue regarding including both people who want to reduce their cigarette intake and people who	The inclusion of both individuals who want to reduce smoking and those who want to maintain abstinence reflects the reality of

want to maintain their quitting. These are qualitatively different outcomes and it is surprising that the intervention has been developed that can address both. Similarly to the point above, can the authors comment on why two behavioural change outcomes are included, and why no smoking frequency criteria is included, as it would be easier for a person to quit if smoking one or two cigarettes a day compared to a smoker of 20 cigarettes a day. The heterogeneity of the sample due to these wide inclusion criteria is somewhat concerning.	smoking cessation in mental health settings, where patients have diverse levels of tobacco dependence and varying motivations to quit. Behaviour change models suggest that smoking reduction can be an important step toward eventual cessation, particularly in populations with complex health needs. By offering tailored support based on individual goals, the intervention remains flexible and patient-centred.<br>While we acknowledge the heterogeneity in smoking behaviours, imposing strict smoking frequency criteria could limit recruitment and reduce the generalisability of findings. Instead, we will collect baseline data on smoking history, including daily cigarette consumption, allowing us to explore how smoking intensity influences engagement and outcomes. This feasibility study will provide insights into whether further refinements, such as stratification by smoking frequency, are necessary for a future definitive trial.
**Reviewer 2: Andrew J. Saxon, University of Washington**	
The manuscript does have a serious flaw in that the discussion section is quite inadequate. Although the design decisions made seem reasonable on their face, several decisions could have been made differently, and an expanded discussion section could address the pros and cons of various design aspects and why the specific methods were chosen. Examples:	Thank you for this feedback. We appreciate your concern about the adequacy of the discussion section. In response, we have made additions to the discussion, specifically addressing various design decisions. We now provide a more detailed explanation of why the specific methods were chosen and how these decisions align with the study's objectives. We hope these revisions better clarify the rationale behind the design and help strengthen the manuscript overall.
While the trial is a randomized test of the intervention, there are apparently no plans to compare outcomes directly between the experimental and control conditions. It is understood that the sample size of the pilot is unlikely to have sufficient power to show statistical separation between the conditions. Many pilot trials do at least calculate effect sizes that can help support the power analysis for the larger, definitive trial. Why is that maneuver not part of the plan for this protocol?	The primary aim of this feasibility study is to assess recruitment, retention, and data collection processes rather than to evaluate the intervention’s effectiveness. While some pilot studies calculate effect sizes, we have not planned direct comparisons between groups because the small sample size increases the risk of unreliable or misleading estimates. Instead, our focus is on assessing the feasibility of a full-scale trial, where an adequately powered sample will allow for a robust evaluation of effectiveness. The feasibility outcomes from this study will

	directly inform the design, including sample size calculations, for a future definitive trial.
It is not clear why nicotine replacement therapy or electronic cigarettes are the only pharmacological options, especially since varenicline is now seen as somewhat superior in efficacy to nicotine replacement therapy. On this topic electronic cigarettes apparently are more controversial among U.S. tobacco treatment experts than they are in the U.K. even though they look good in randomized trials as a cessation pharmacotherapy and probably pose less health risks than combustible tobacco. Nevertheless, they probably have more health risks than nicotine replacement so the decision to use them should be justified. Also, more detail about the nicotine replacement would be helpful. Will the protocol allow combination of a long-acting form of replacement (i.e. transdermal nicotine) with a short-acting form (e.g. gum or lozenge)?	The trial was designed with necessary alignment to UK policy and practice. As the reviewer points out, these vary internationally. At the time the protocol was written, the UK National Institute for Health and Care Excellence (NICE) had issued guidance for all mental health Trusts to provide nicotine replacement therapy to their patients. At the time, Varenicline was still largely contested in mental health contexts in the UK, and hardly ever prescribed (similar to Bupropion). We could be confident that NRT would be available to all patients in all Trusts, and as such restricted the option to this.<br><br>Irrespective of the above, in the UK, Varenicline (Champix) also happens to have been unavailable on the market between 2021 and 2024 (due to the presence of nitrosamines).<br><br>Regarding e-cigarettes, it is true that the UK has got a rather more favourable outlook on their smoking cessation/harm reduction potential, especially for multiply disadvantaged groups, than other countries. Indeed, new NICE guidance explicitly recommends their use for smoking cessation, including in mental health populations: https://www.nice.org.uk/guidance/ng209<br><br>This has been added to the manuscript, both in the Strength and Limitations summary section at the start of the manuscript, and in the Limitations section in the discussion.
Many patients admitted to inpatient psychiatric units suffer from psychosis and/or mania, yet the protocol has no instruments to assess such symptomatology. Why not? Such syndromes could be affected by tobacco cessation treatments.	We appreciate this comment, and agree that it would have been interesting to assess the potential impact of our intervention on symptoms of psychosis. However, this was not a priority outcome for us, and the list of secondary outcomes is already long, with some concerns relating to burden of assessment. Importantly, administering clinical instruments to assess these diagnosis-specific outcomes would have required expert researchers (psychiatrically

	trained/experienced), and this would have posed substantial practical issues.
Many trials that study a behavioral intervention use actual recordings of treatment sessions to assess fidelity. This protocol relies mainly on self-report by the clinicians delivering the intervention for fidelity assessments. What prompted the decision not to record the sessions? Additionally, in regards to line 332, What would "random fidelity checks" consist of?	The decision not to record sessions was based on findings from a very small pilot study to test the viability of the protocol and materials. We found that practitioners and patients were not comfortable with session recordings during and shortly after discharge, primarily due to logistical and ethical considerations, such as concerns around patient confidentiality, consent, and the resources required for reviewing these. While we acknowledge that session recordings are very valuable for fidelity assessments, these factors led us to rely on self-report by clinicians in this protocol. We will consider incorporating recordings in future studies if feasible with proper consent but highlight that our study population is exceptionally vulnerable, consisting of acutely unwell people, many of whom have psychosis. This has been added to the discussion as a limitation.
Since breath carbon monoxide is the primary outcome measure and since new technologies have the ability to do remote carbon monoxide testing and since most of the interventions will be delivered remotely anyway, why was remote breath carbon monoxide testing not considered? It would likely reduce the amount of missing data in regard to the primary outcome. On this topic the manuscript alludes to the fact that the optimal breath carbon monoxide level to determine abstinence may be uncertain and that the chosen threshold of 10 parts per million may be too high and might need to be lowered. What, then, led to the decision to choose this somewhat high threshold?	The protocol was finalised in 2021, at which point we were not aware of these technologies but agree that this would be most useful to explore, should a full trial go ahead. The present feasibility trial is now closed.
An additional important point is that person centered, non-stigmatizing language should be used throughout the protocol. "Patients who smoke tobacco" rather than "smokers" is more appropriate. The terms "smoker" or "smokers" should be completely eliminated from the manuscript.	This has been corrected throughout the manuscript.
The acronyms "PIS" and "APEASE" require definitions.	We have corrected this.

VERSION 2 - REVIEW

**Reviewer**	**1**
**Name**	**OConnell, Nicola.**
**Affiliation**	**Health Service Executive**
**Date**	**23-May-2025**
**COI**	

The authors have taken on board reviewers' suggestions. It may be the case here that the authors assume people in other jurisdictions will understand how NHS mental health trusts work and UK national policies. It is wise to assume most readers won't and clarify where possible/relevant.

**Reviewer**	**2**
**Name**	**Saxon, Andrew J.**
**Affiliation**	**University of Washington**
**Date**	**15-May-2025**
**COI**	

This manuscript, which describes the protocol for a randomized, pilot feasibility trial on a an intervention to help patients who smoke tobacco and are admitted to a smokefree psychiatric inpatient unit and must stop tobacco use during the inpatient stay to extend tobacco abstinence into the post-discharge phase, has been revised with some success according to the reviewers' and editor's suggestions.

However, still not included in the limitations section is the fact that many participants in the trial have psychosis or bipolar disorder, yet the impact of tobacco cessation efforts on the status of these important psychiatric conditions is not assessed during the trial.

Also, the revisions resulted in two areas where the writing has become mangled.

Line 58: This sentence is now grammatically incorrect.

Lines 84-86: Rewrite this bullet point which has become unreadable with the revisions.

VERSION 2 - AUTHOR RESPONSE

Reviewer: 1
Dr. Nicola. OConnell, Health Service Executive
Comments to the Author:
The authors have taken on board reviewers' suggestions. It may be the case here that the

authors assume people in other jurisdictions will understand how NHS mental health trusts work and UK national policies. It is wise to assume most readers won't and clarify where possible/relevant.

Thank you for this helpful comment. We agree that international readers may not be familiar with how NHS Mental Health Trusts operate or with the structure of national policy implementation in the UK.

To address this, we have made the following clarifications in the manuscript:

1. ***In the "Study setting" section (Methods and Analysis), we have added a brief explanation of what NHS Mental Health Trusts are and how they function within the broader NHS framework in England.***

Additionally, in the "Discussion" section, we have listed the UK-specific policy context preventing generalisability as a limitation, to support better international understanding.

Reviewer: 2
Dr. Andrew J. Saxon, University of Washington
Comments to the Author:
This manuscript, which describes the protocol for a randomized, pilot feasibility trial on a an intervention to help patients who smoke tobacco and are admitted to a smokefree psychiatric inpatient unit and must stop tobacco use during the inpatient stay to extend tobacco abstinence into the post-discharge phase, has been revised with some success according to the reviewers' and editor's suggestions.
However, still not included in the limitations section is the fact that many participants in the trial have psychosis or bipolar disorder, yet the impact of tobacco cessation efforts on the status of these important psychiatric conditions is not assessed during the trial.

Thank you for this important observation. We agree that this is a relevant limitation and have now explicitly acknowledged that while the study population includes individuals with severe mental illness, including psychosis and bipolar disorder, we do not assess the specific impact of the intervention on these conditions. We have revised the limitations section of the discussion accordingly, adding a statement to highlight that condition-specific psychiatric outcomes are not captured and recommending their inclusion in future studies.

Also, the revisions resulted in two areas where the writing has become mangled.
Line 58: This sentence is now grammatically incorrect.

Thank you, this has been corrected.

Lines 84-86: Rewrite this bullet point which has become unreadable with the revisions.

Thank you, this has been corrected.
